# Atomistic Simulation of Loading Direction-Dependent Brittle-to-Ductile Transition in a ⟨110⟩-Oriented B2-FeAl Nanowire Under Bending

**DOI:** 10.3390/nano16181172

**Published:** 2026-09-17

**Authors:** Zhaozhao Wei

**Affiliations:** School of Mechanical and Automation Engineering, Wuyi University, Jiangmen 529020, China; wei_zhaozhao@wyu.edu.cn

**Keywords:** B2-FeAl nanowire, bending deformation, loading direction, molecular dynamics simulation

## Abstract

Molecular dynamics simulation was employed to investigate the effect of loading direction on the bending behavior of a B2-FeAl nanowire. The simulations were carried out with different loading directions of [001], [112], [111] and [110] at 300 K. The results show that the FeAl nanowire yields by nucleation of dislocations in all cases, which is independent of the loading direction. However, various loading directions result in different mechanical responses and failure mechanisms. Under [001]- and [112]-bending, the nanowire fails in a brittle manner, whereas changing the loading direction to [111] and [110] directions produces significant plastic deformation with large bending curvature. The brittle-to-ductile transition with respect to loading directions is related to the dislocation characteristics and their cross-slip propensity. Dislocation analysis reveals that [001]- and [112]-bending generate edge-dominant dislocations with limited capability of cross-slip, thereby resulting in stress concentration and subsequent brittle fracture, whereas [111]- and [110]-bending deformation is mediated by slip of dislocations with predominant screw characteristics, whose cross-slip process effectively relaxes stress localizations and sustains uniform plastic deformation. Moreover, a physical model incorporating bending-induced geometric constraints is established to quantify the evolution of dislocation characteristics with respect to loading directions, providing an efficient theoretical framework to rationalize the bending plastic anisotropy of the nanowire.

## 1. Introduction

Metal nanowires have attracted considerable attention owing to their excellent mechanical, electrical and optical properties and potential applications in nano/micro electromechanical systems (NEMS/MEMS) [1,2,3]. Particularly, the nanowires may undergo severe and repeated bending deformations in the manufacture and application of these nanodevices. Therefore, improving the endurance and reliability of the nanodevices requires a comprehensive understanding of the mechanical properties of nanowires related to bending loads. Over the past decades, considerable efforts have been devoted to investigating the bending behaviors of metal nanowires. Based on a proper understanding of deformation behaviors and failure modes, the mechanical properties of nanowires under bending conditions could be significantly improved. For instance, the introduction of a fivefold twin structure leads to a remarkable enhancement in the elastic modulus and strength of Ag nanowires due to effective grain-boundary hardening [4]. Moreover, in situ experiments have also unveiled some unique mechanical properties of metal nanowires upon bending, including abrupt elastoplastic transitions [5] and the superelasticity of the ultra-large bending angle [6].

Despite the above progress in experimental results, performing mechanical testing of nanowires remains challenging due to the limited spatiotemporal resolution related to the rapid changes in microstructures under loading. In this context, molecular dynamics (MD) simulation plays a key role in exploring the deformation behavior of nanowires. Based on detailed MD simulations, it has been shown that the plastic deformation mechanism of metal nanowires under bending is highly sensitive to their crystallographic orientations and microstructures. Under bending, Ni nanowire oriented in [112] axial direction deforms predominantly by slip of extended dislocations as manifested by uniform plastic deformation, whereas premature fracture was observed in [111] and [010] orientations due to extensive dislocation pile-ups [7]. It has also been found that fivefold twins form in a [001]-oriented Cu nanowire upon bending, while such a twin structure was absent in a nanowire with [110] orientation, where plastic deformation was dominated by dislocation slip [8]. Microstructural design has been shown to be an effective approach for tailoring the mechanical properties of nanowires. The presence of coherent twin boundaries can further modulate the competition between dislocation slip and deformation twinning, allowing for a tunable balance between strength and ductility [9,10]. Some extraordinary deformation modes have also been uncovered by MD simulations. Bent Ag nanowires with diameters around 5 nm undergo sequential phase transformations from FCC to BCC to HCP structures, allowing for the accommodation of bending strain via structural change rather than dislocation motions [11]. For [110]-oriented Cu and Au nanowires, bending deformation can lead to the formation of wedge-shaped twins, which exhibit spontaneous pseudoelastic recovery upon unloading [12]. Similar pseudoelasticity was also observed in *α*-Fe nanowires, which is driven by high interfacial energy stored in the nonconventional {110} twin boundaries [13,14]. In addition, MD studies consistently demonstrate a strong size effect on the mechanical properties of nanowires subjected to bending. It is shown in some FCC nanowires that the elastic modulus and yield strength increase with decreasing diameter below a critical size (typically ~30 nm), which is attributed to the enhanced surface effect with the decrease insample sizes in diameters [15,16].

Apart from the above intrinsic structural factors, extrinsic deformation conditions, including temperature, strain rate and loading direction, also play a crucial role in the mechanical performance of metal nanowires. Elevated thermal conditions may weaken the overall bending response of nanowires by lowering their elastic modulus and yield strength, which has been well validated in existing studies [16,17]. High strain rates tend to shorten the plastic regime and facilitate brittle fracture in nanomaterials [18]. Furthermore, loading direction also serves as one important factor that fundamentally governs the bending properties and deformation mechanisms of nanowires. While the twinning-mediated bending plasticity in FCC nanowires is unaffected by loading direction [10,12], it is strongly influenced in BCC nanowires when bent along different directions [19,20]. Changes in bending direction drastically alter the internal stress distribution within nanowires. The resulting anisotropy directly governs the activation of preferred slip systems, thereby determining the dominant deformation mode and mechanical properties of nanowires during bending.

As can be seen, most of the above studies on the bending behavior of nanowires have focused on pure metals, whereas little attention has been paid to alloy nanowires. FeAl is an intermetallic ordered alloy with a B2 structure, where Fe and Al atoms occupy the corners and the body center of a BCC lattice, respectively. Owing to the high strength, excellent corrosion resistance and low density, FeAl has attracted considerable attention and found widespread applications in high-temperature structural components and protective coatings under extreme service conditions. However, B2-structured intermetallic compounds are generally brittle at ambient temperature, which severely hampers their further applications [21]. As a typical nanostructured counterpart, one-dimensional nanowires are expected to improve the strength and ductility of such B2-structured intermetallics. For instance, the B2-NiAl nanowire has been reported to show low-temperature superplasticity behavior with a failure strain of ~700% at 700 K [22]. In addition, B2-CuZr nanowire has been reported to achieve up to 45% recoverable plastic strain via stress-induced martensitic transformation [23]. A recent study has also found that B2-FeAl nanowires with different axial orientations exhibit distinct bending behavior [24], in which the nanowire oriented in the ⟨110⟩ axial direction shows excellent plasticity and fracture toughness under bending conditions. Further investigation involving the influence of loading directions on such bending plasticity is of great interest, as it is related to the reliability and manufacturability of flexible nanodevices [25,26]. In view of this, detailed MD simulations have been conducted on a ⟨110⟩-oriented B2-FeAl nanowire upon bending along different loading directions in this study. Emphasis has been placed on the deformation mechanisms, defect characteristics and fracture behavior with respect to the loading directions.

## 2. Simulation Details

**Figure 1:**
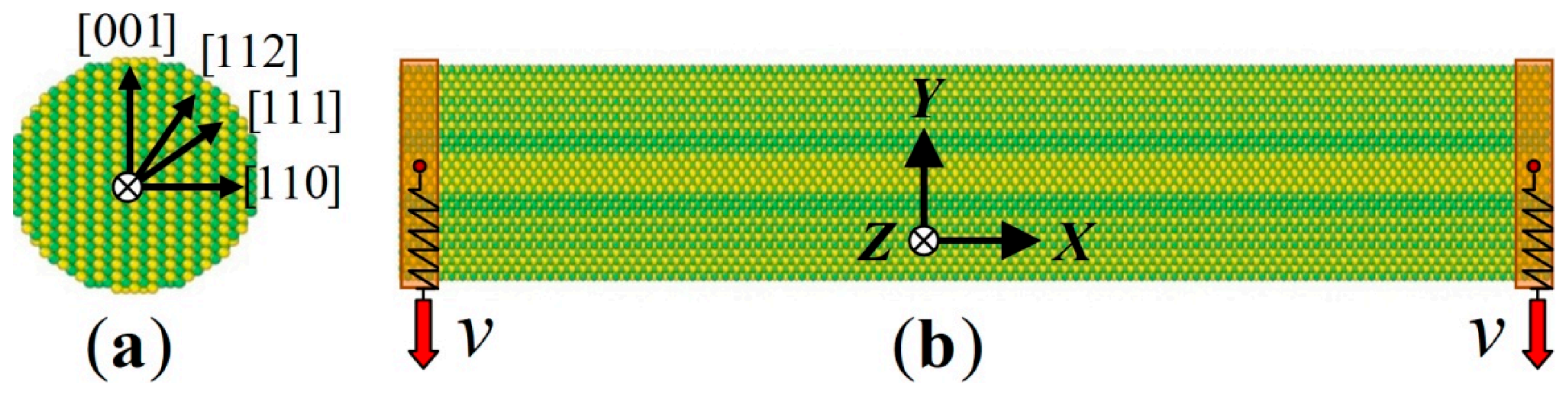
Schematic illustration of sectional geometry (**a**) and the simulation setup for bending (**b**) of the ⟨110⟩-oriented B2-FeAl nanowire.

In this work, a ⟨110⟩-oriented B2-FeAl nanowire is adopted as the model system, as shown in Figure 1. The FeAl nanowire possesses a circular cross-section in order to preclude the cross-sectional effect on the bending behavior, as presented in Figure 1a. The nanowire was initially created along the X-$\left[1\bar{1}0\right]$, Y-[001] and Z-[110] directions, which has dimensions of 44.4 nm × 5.9 nm × 5.9 nm with an aspect ratio of about 7.5, containing a total number of 87885 atoms. The X axis of the simulation region is parallel to the axial direction of the nanowire, and the Y axis denotes the loading direction of bending deformation. The interatomic interaction between Fe and Al atoms was described by the embedded atom method (EAM) potential developed by Mendelev et al. [27]. This potential could reasonably describe the fundamental physical properties of B2-FeAl alloy, such as lattice constant, cohesive energy and elastic constants, which has been successfully applied in studies concerning mechanical performance and dislocation analysis of FeAl alloys [28,29]. A simulation timestep of 1 fs was adopted, and free boundary conditions were applied in X, Y and Z directions for all simulations. Before loading, the initial configuration of the nanowire was energy-minimized using the conjugate gradient to achieve a stable structure with minimum potential energy. Subsequently, the nanowire was relaxed for 50 ps within the canonical (NVT) ensemble at a temperature of 300 K to reach thermodynamic equilibrium.

In order to apply bending deformation, a spring with a stiffness of 30.0 N/m was coupled to the atomic layers with a thickness of 0.5 nm at each end of the nanowire, as illustrated in Figure 1b. Bending was loaded by moving the free ends of the springs at a constant velocity of 0.005 nm/ps along the prescribed loading direction; meanwhile, the arising momentum was eliminated by subtracting the center-of-mass velocity of the system. To investigate the influence of the loading direction on the bending behavior, bending moments were applied to the nanowire along directions of [001], [112], [111] and [110], respectively, which are nearly uniformly distributed in the <110> zone, as illustrated in Figure 1a. During the bending deformation, the spring force (*F*) and displacement of its free end (*d*) were recorded to construct *F-d* curves, which characterize the mechanical response of the nanowire under bending. The comparison of these obtained *F-d* curves provides information on the bending behavior of the B2-FeAl nanowire with respect to different loading directions.

All MD simulations were performed using the Large-scale Atomic/Molecular Massively Parallel Simulator (LAMMPS) package [30]. The atomic configurations were visualized by using the post-processing software OVITO basic (Open Visualization Tool, version 3.14.1) [31]. The microstructural evolution during bending deformation was analyzed by using the common neighbor analysis (CNA) method [32]. The dislocation characteristics were analyzed using the dislocation extraction algorithm (DXA) [33]. Furthermore, the local deformation state within the nanowire was also characterized using the atomic local shear strain analysis [34].

## 3. Results

### 3.1. Mechanical Response

**Figure 2:**
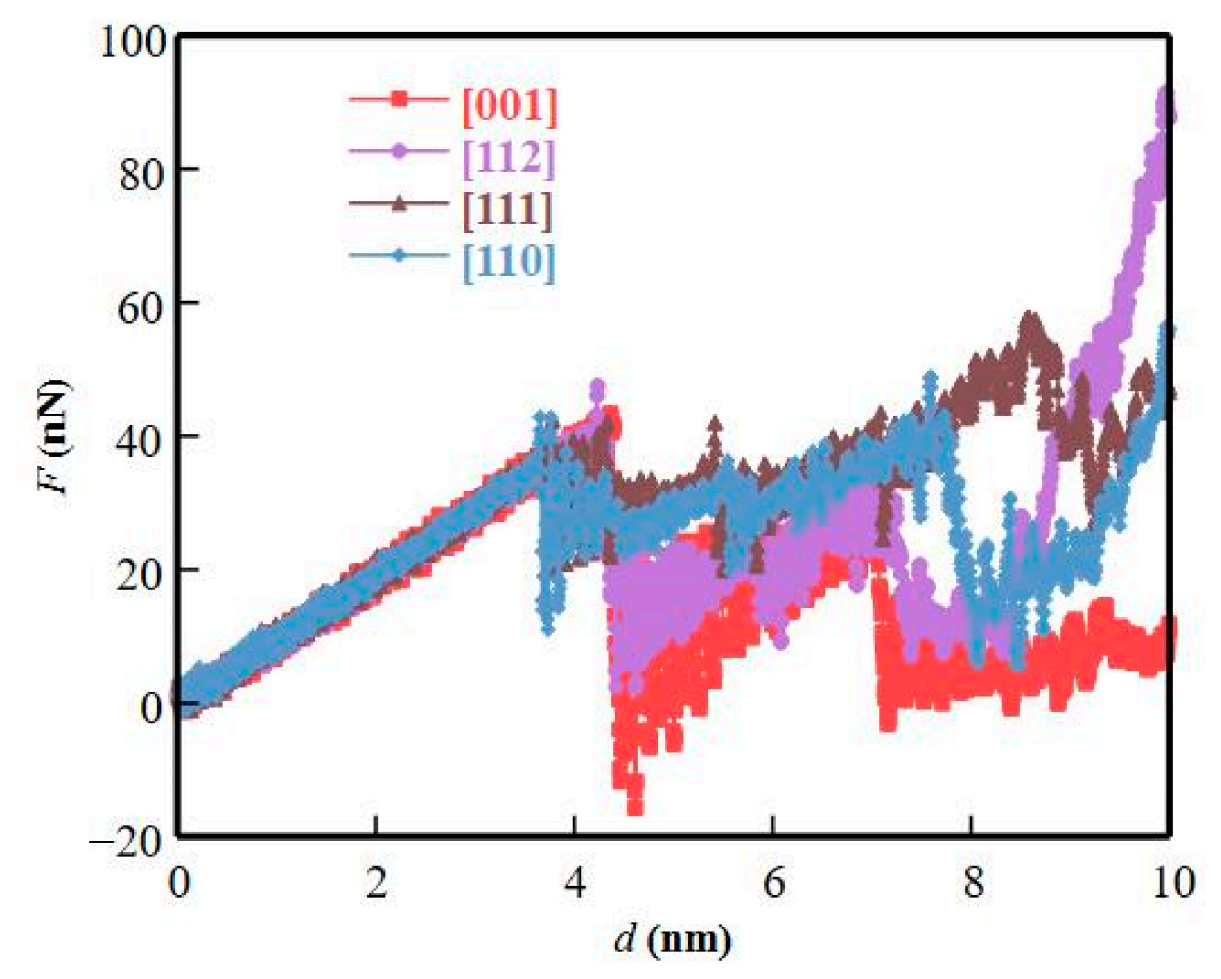
*F-d* curves of the ⟨110⟩-oriented B2-FeAl nanowire under bending along different loading directions.

MD simulations of the ⟨110⟩-oriented B2-FeAl nanowire under bending with different loading directions of [001], [112], [111] and [110] has been performed, respectively. The deformation state is characterized by the *F-d* curves within the framework of the ideal Euler–Bernoulli beam theory. Figure 2 presents the corresponding *F-d* curves related to different bending directions. It can be seen in the figure that the bending deformation primarily includes initial elastic and subsequent plastic regimes for all loading directions. In the elastic stage, the loading force increases linearly with the displacement, showing consistency with the linear elastic theory of a thin beam under bending. Notably, the elastic regimes for different loading directions overlap. According to the bending elasticity theory [35], the elastic modulus of the nanowire is proportional to the bending stiffness associated with the force-to-displacement ratio of *F*/*d*. It may be deduced that the elastic modulus of the FeAl nanowire in bending is independent of the loading direction.

At the end of elastic stage, occurrence of an abrupt drop signifies yielding of the nanowire and the onset of plastic deformation. The yield strength of nanowire can be quantified by the maximum bending stress *σ*_max_ in the nanowire, which is a function of the maximum bending moment as well as the cross-section, and may be written as σmax=MmaxymaxI, where *M*_max_ is the maximum bending moment, *I* denotes the area moment of inertia and *y*_max_ is the maximum *y*-position from the neutral axis within the cross-section. The maximum bending moment is further calculated as *M*_max_ = *F*_max_*L*, where *F*_max_ represents the peak force and *L* is the force lever arm. For a given nanowire geometry, the yield strength measured by the *σ*_max_ is therefore proportional to the peak force. Consequently, the peak values of force in the *F-d* curves characterize the yield strength of a defect-free nanowire under bending and serve as an identifiable indicator for the onset of yielding. Accordingly, different loading directions result in various yield strengths of the nanowire. The [110]-bending shows the lowest yield strength, followed by an increase in the order of [111]-, [112]- and [001]-bending. This plastic anisotropy in yield strength may be attributed to the influence of loading direction on the crystallographic orientation of the bent surfaces. As the loading direction changes, the orientation as well as the atomic arrangement of these bent surfaces vary significantly, resulting in different local stress and nucleation energies barrier of dislocation. The difference in surface stress states thus leads to a strong dependence of yield strength on the loading direction, given that the dislocations preferentially nucleate from the surface of the nanowire due to its high surface energy.

Further, it can be seen that the profiles of the *F-d* curves in the plastic stage vary significantly as the loading direction changes from [001] to [110], indicating a close relationship between the bending plasticity and the loading direction. For the [001] and [112] loading directions, the force drops abruptly to a low level at the yielding point, and a second drop of force can be observed at larger deformation, suggesting fracture of the nanowire. In contrast, there is a plateau after the yield drop of force in the *F-d* curves for both cases of [111]- and [110]-bending, which implies uniform plastic deformation within the nanowire. The force remains relatively constant during the plateau region covering a range of displacement close to 4 nm, which is followed by a second drop of force that corresponds to ductile failure. The force and displacement at the second drop correspond to the maximum bending force and bending deformation that the nanowire can sustain before failure, characterizing, respectively, the bending strength and plasticity of the nanowire. It is revealed that the nanowire, upon [111]-bending, exhibits the highest bending strength in addition to the bending plasticity as compared to the other loading directions. The above results clearly suggest that the bending behavior of the B2-FeAl nanowire is strongly dependent on the loading direction, which will be discussed as follows.

### 3.2. Deformation Behavior and Failure Mode

**Figure 3:**
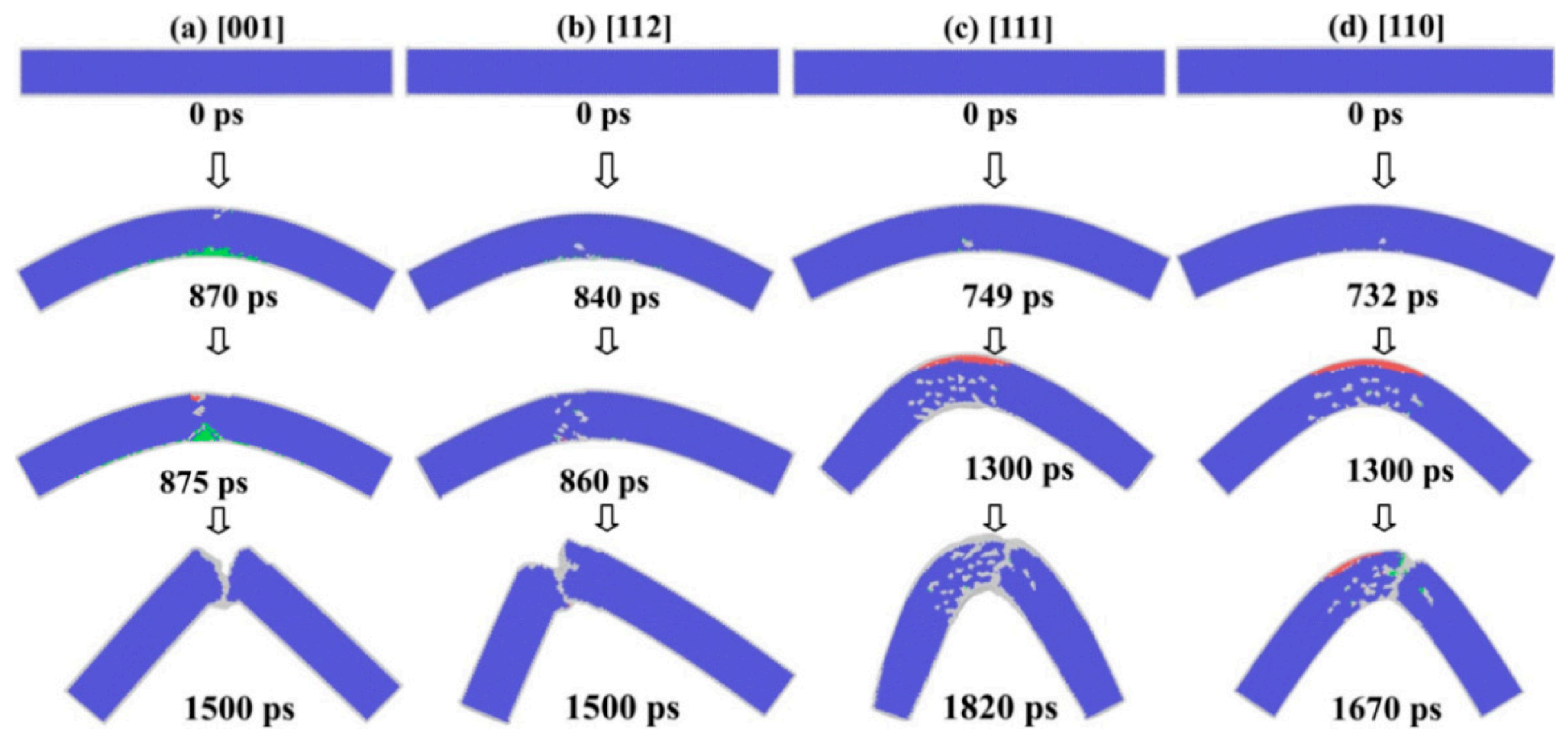
Atomic configurations of the ⟨110⟩-oriented B2-FeAl nanowire upon bending along (**a**) [001], (**b**) [112], (**c**) [111] and (**d**) [110] directions. Colors denote local crystal structures: blue—BCC, green—FCC, red—HCP, white—unknown.

To understand the difference in mechanical response under bending, the microstructural evolutions of the FeAl nanowire have been visualized and analyzed using OVITO. Figure 3 presents the atomic configurations of the nanowire under bending at various deformation stages related to different loading directions. The CNA method is employed to determine the crystal structure during deformation. Since the CNA does not distinguish the atom types, the B2 ordered lattice is identified as a BCC structure, as represented by the blue atoms in the figure. As can be seen, the yielding of a nanowire occurs by nucleation of dislocations at the free surfaces in all cases. Accordingly, the yielding mechanism of the FeAl nanowire is insensitive to the loading directions.

However, the change in loading directions leads to distinct deformation behavior as well as different failure modes. Under [001]-bending, the yielding event is immediately followed by nucleation of a sharp crack from the upper surface of the nanowire, as shown in Figure 3a. The tensile stress produced in the upper surface by bending promotes rapid growth of the crack along the direction at an angle of about 45° with the nanowire axis. When the growing crack reaches the opposite surface at a small loading displacement, the nanowire fails abruptly and forms a flat fracture surface without exhibiting significant plastic deformation. Similarly, the FeAl nanowire exhibits comparable deformation characteristics when bent along the [112] direction. With the generation of a small number of dislocations, a crack nucleates at a small bending deformation and propagates rapidly inwards, ultimately causing brittle fracture of the nanowire, as depicted in Figure 3b. These observations clearly suggest that the ⟨110⟩-oriented B2-FeAl nanowire fails in a brittle manner under bending along the [001] and [112] directions.

In contrast to the brittle fracture in [001]- and [112]-bending, more dislocations are generated after yielding as deformation increases in [111]- and [110]-bending, as shown in Figure 3c,d. Since the initial configuration of the FeAl nanowire is free of defects, the dislocations once nucleated glide rapidly on their respective slip plane towards the nanowire interior. The continuous glide of multiple dislocations results in the formation of a force plateau, as observed in the *F-d* curves. Following the plateau region, further deformation promotes the accumulation of dislocations within the nanowire, inducing stress concentration and subsequent necking in the later deformation stage of bending. Accordingly, the nanowire undergoes substantial plastic deformation and ultimately fails by ductile fracture. Interestingly, it is noted that the FeAl nanowire under [111]- and [110]-bending deforms uniformly and plastically into a smooth arc shape with a large bending angle, which is unusual for a brittle material like a B2-FeAl intermetallic. Even when the bending angle is close to 180°, the nanowire does not show complete separation, highlighting its excellent bending plasticity and ductility, as compared to its bulk counterparts.

**Figure 4:**
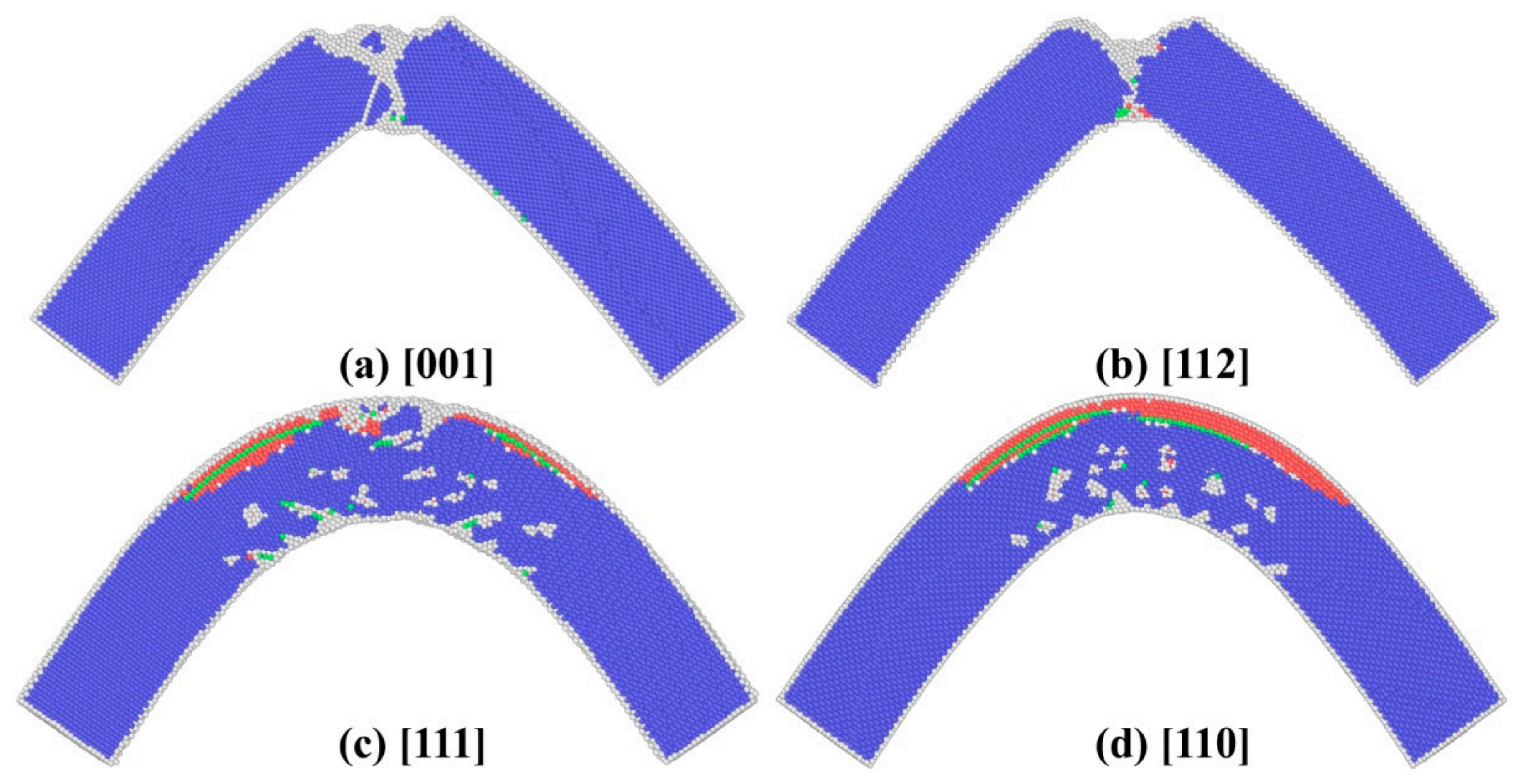
Atomic configurations of the ⟨110⟩-oriented B2-FeAl nanowire at simulation time of 40 ps upon bending along (**a**) [001], (**b**) [112], (**c**) [111] and (**d**) [110] directions. Colors denote local crystal structures: blue—BCC, green—FCC, red—HCP, white—unknown.

It is noted that the deformation results above were obtained from simulations performed at a single loading velocity. Since loading velocity strongly influences the fracture mode, it is essential to verify whether the observed brittle-to-ductile transition is dependent on the chosen loading velocity. Thus, we have carried out additional bending simulations using a relatively larger loading velocity of 0.2 nm/ps, and the corresponding results are presented in Figure 4.

It is demonstrated that even under a relatively large loading velocity, the nanowires still retain considerable bending plasticity upon [111]- and [110]-bending, while [001]- and [112]-bending continue to display similar brittle fracture characteristics. It might be assumed that the observed brittle-to-ductile transition related to loading directions is not an artifact of the loading velocity.

### 3.3. Analysis of Dislocation

#### 3.3.1. Dislocation Configuration

**Figure 5:**
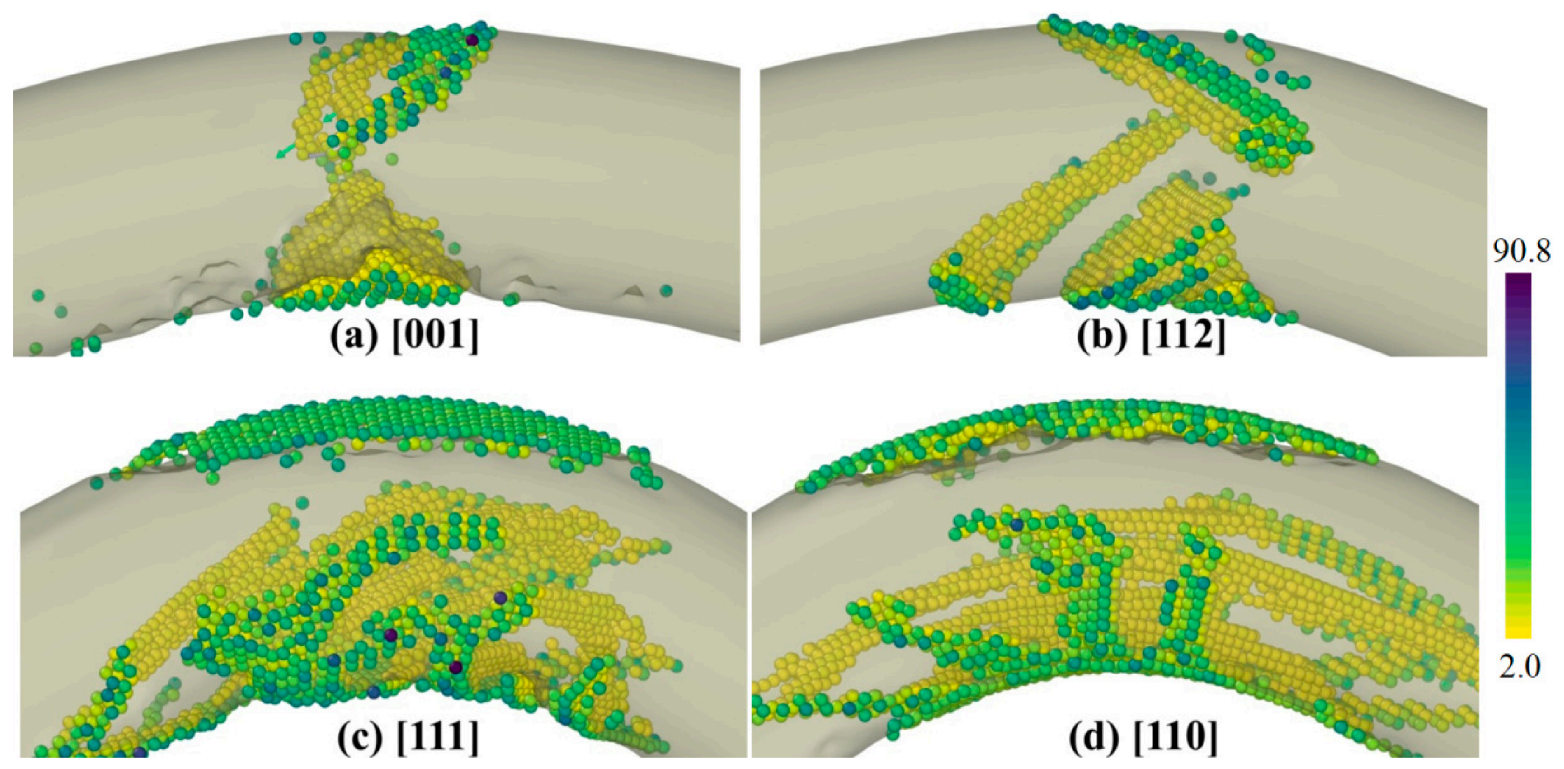
Dislocation configurations of the ⟨110⟩-oriented B2-FeAl nanowire upon bending along (**a**) [001], (**b**) [112], (**c**) [111] and (**d**) [110] directions; the atoms are colored according to their CSP, and only the atoms with high CSP are visible.

The above results demonstrate that the dominant yielding mechanism of the ⟨110⟩-oriented B2-FeAl nanowire involves dislocation nucleation for all loading directions. The dislocations serve as the carriers of bending plasticity, particularly for the cases of [111]- and [110]-bending. Using the DXA analysis, we found that all generated dislocations have Burgers vectors of 1/2⟨111⟩, which is commonly observed in B2 alloys. The dislocation configuration could be identified by the centrosymmetry parameter (CSP), as presented in Figure 5. It can be clearly seen that the number of dislocation lines generated by [001]- and [112]-bending is much lower than that for [111]- and [110]-bending, corresponding to the brittle fracture occurring in the former two cases and good ductility in the latter two. The dislocation lines are initially curved upon nucleation, and subsequently align themselves to a straight configuration under further deformation. Notably, these dislocation lines appear to be perpendicular to the loading direction. This arrangement of dislocations is attributed to the non-uniform strain field produced by bending. While a prominent strain gradient is introduced along the loading direction, a uniform strain field forms along the width direction of the nanowire. Since the strain field of a dislocation along its line direction is uniform, the dislocations tend to align themselves parallel to the neutral plane and consequently adopt an orientation perpendicular to the loading direction.

**Figure 6:**
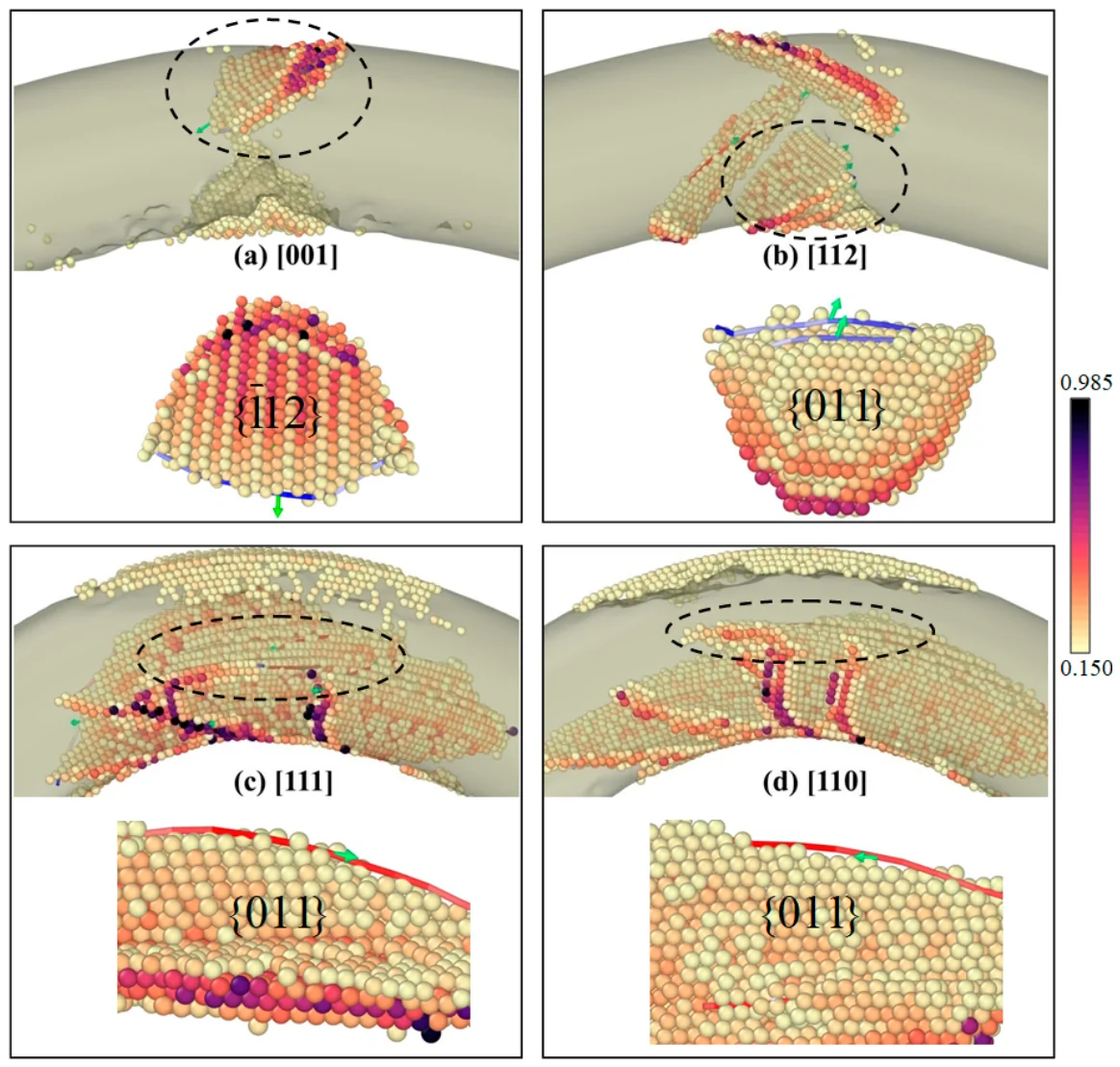
Dislocation slip planes in the ⟨110⟩-oriented B2-FeAl nanowire upon bending along (**a**) [001], (**b**) [112], (**c**) [111] and (**d**) [110] directions; atoms are colored by their atomic local shear strain, and only those with strain larger than 0.15 are visible. The insets show the enlarged images of the region enclosed by dashed circle.

The atomic local shear strain, defined by Shimizu [34] can be used to detect the slip planes of the dislocations and visualize their movements inside the nanowire. As shown in Figure 6, atoms are colored by their local shear strain, and only the atoms with a strain larger than 0.15 are visible. In this way, the slip planes of dislocations could be clearly identified, as further illustrated by the enlarged insets in the figure. Detailed crystallographic analysis reveals that the slip plane of dislocations in [001]-bending is {112}, as shown by the enlarged image in Figure 6a, which is more commonly observed at low temperature and rarely reported during room-temperature deformation [36,37]. On the other hand, the {011} slip plane is identified in the cases of [112]-, [111]-, and [110]-bending, as evidenced by the enlarged insets in Figure 6b–d, which is consistent with the experimental observations and simulation results [38,39,40,41,42]. The observation of the {112} slip plane in [011]-bending might stem from the strong surface effects at the nanoscale, which may lower the nucleation barrier for {112} slip. Moreover, the 1/2⟨111⟩{112} slip system exhibits the maximum Schmid factor of 0.471 for a ⟨110⟩-oriented B2 nanowire, which further increases the likelihood of its activation based on the Schmid criterion.

**Figure 7:**
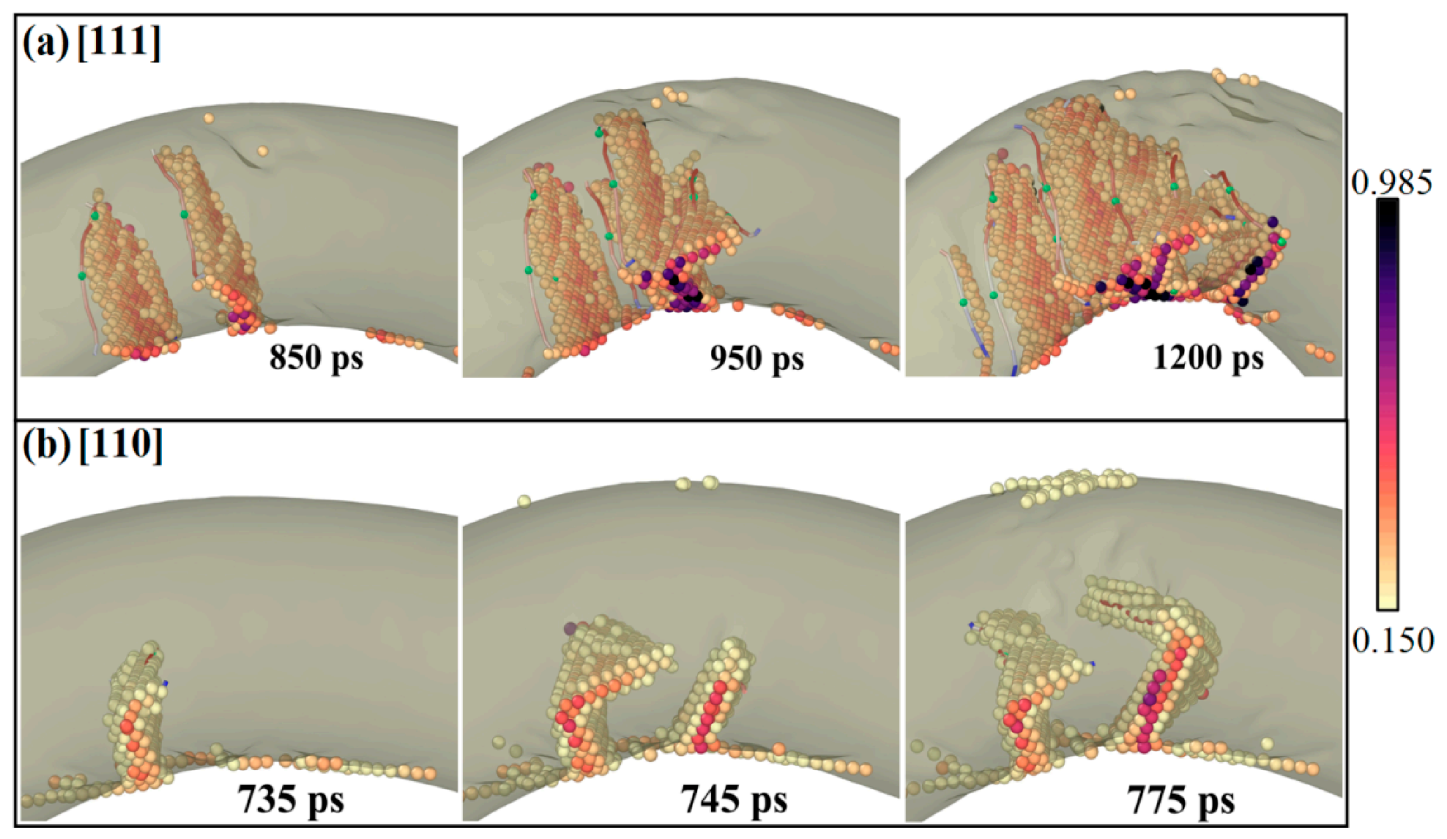
Cross-slip events of dislocation in the nanowire upon bending along (**a**) [111] and (**b**) [110] directions; atoms are colored by their atomic local shear strain and only those with strain larger than 0.15 are visible.

Further, the slip trajectories of dislocations can be effectively identified by the color contrast related to the local shear strain, where light yellow denotes low shear strain and dark red corresponds to higher strain value. Given the limited dislocation activity in [001]- and [112]-bending, the analysis of dislocation pathways focuses primarily on the cases of [111]- and [110]-bending. According to the dislocation traces, it appears that the dislocations nucleate and glide subsequently on a set of parallel slip planes, as depicted in Figure 6c,d. These dislocations tend to be uniformly distributed in the nanowire without forming considerable entanglements due to their mutual interactions. This is different from the complex dislocation network commonly observed in uniaxial deformation [43,44,45,46,47], which highlights the significant role of the loading mode on the dislocation configuration. The trace analysis of dislocations also indicates that cross-slips occur as the dislocations move into the nanowire. Figure 7 provides clearer visualization of the cross-slip processes by sequential snapshots of the dislocation slip planes. The cross-slip processes enable the dislocations to glide on the planes with the maximum resolved shear stress, thereby maintaining stable plastic flow in the nanowire [48].

#### 3.3.2. Dislocation Types

**Figure 8:**
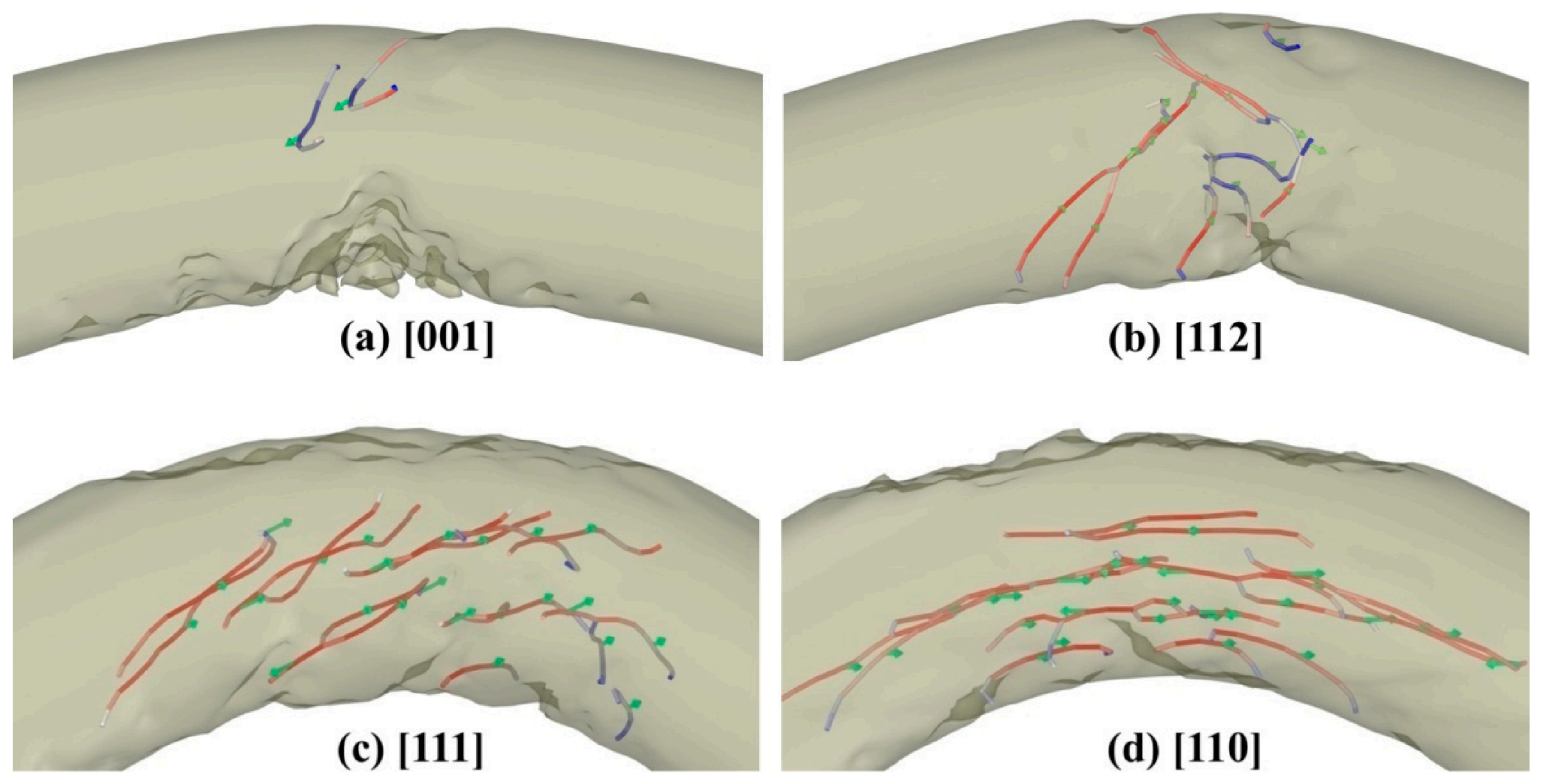
Dislocation lines in the ⟨110⟩-oriented B2-FeAl nanowire upon bending along (**a**) [001], (**b**) [112], (**c**) [111] and (**d**) [110] directions; green arrows denote the Burgers vector, and blue and red lines denote edge and screw dislocation, respectively.

The dislocation types dictate their slip propensity, which directly governs the plastic deformation of the nanowire. Accurate identification of the dislocation type provides a deeper understanding of the relationship between the microstructure and the mechanical properties. Based on the DXA analysis, Figure 8 presents the types of those nucleated dislocations under various loading directions. The results show significant dependence of the dislocations type on the loading direction. In [001]-bending, the plastic deformation is dominated by slip of edge dislocations, as illustrated by the blue lines in Figure 8a. As the loading direction shifts to [112], mixed dislocations are generated, as manifested by the mixture of blue and red lines in Figure 8b. When the loading direction is further changed to [111] and [110] directions, screw-dominant mixed dislocations prevail in the bending deformation, as indicated by the red lines in Figure 8c,d.

**Figure 9:**
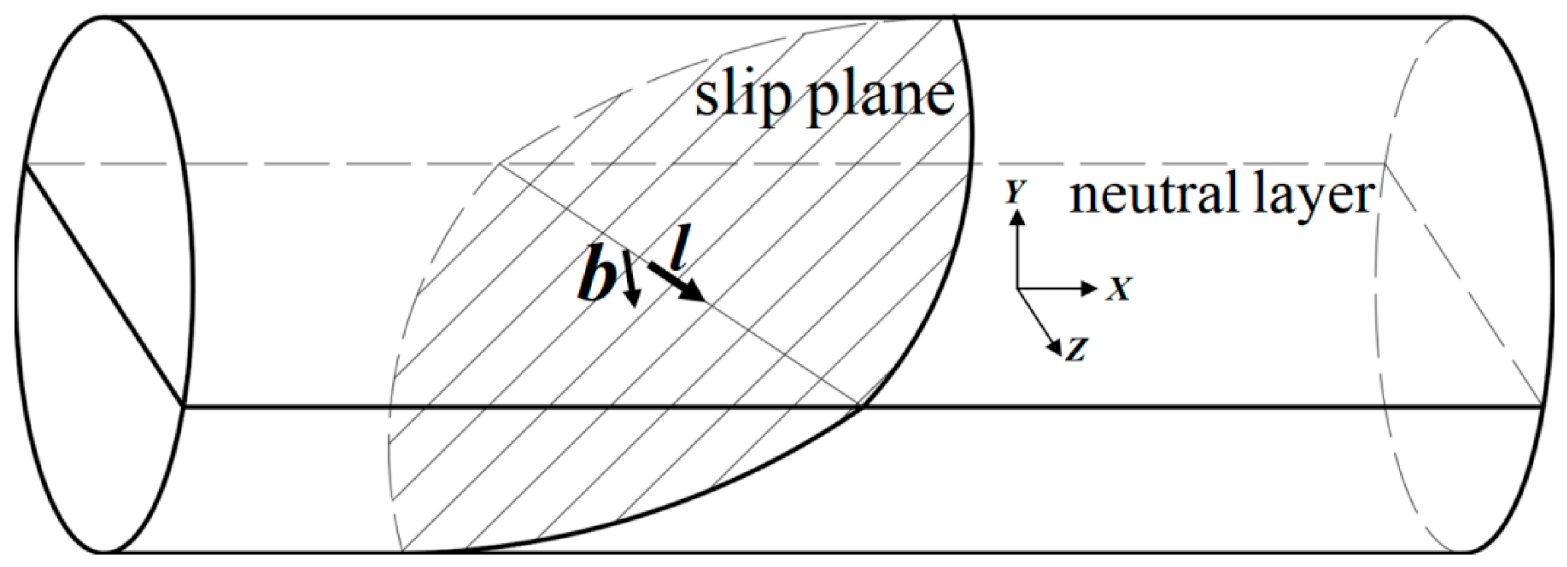
Scheme of the geometric method for determining the dislocation type based on bending geometry constraints.

Although the DXA method readily identifies the dislocation types, here we employ a simple method based on the geometric constraints in bending to further analyze the dislocation characteristics. Since the dislocations preferentially nucleate from the free surfaces due to the required energy condition, the nucleation site could be determined by the intersection between the active dislocation slip plane and the free surface. Additionally, the above results demonstrate that the dislocations adopt a straight configuration and align parallel with the neutral plane of the nanowire. Therefore, the line vector ***l*** of a dislocation could be characterized by the intersection between the slip plane and the neutral plane, as illustrated in Figure 9. The intersection can be determined by the cross product of the normal vectors of the respective planes. In this manner, the dislocation type may be characterized by examining the angle between its Burgers vector ***b*** and the line direction ***l***. Based on the dislocation slip planes and the Burgers vectors, the determination of dislocation types is summarized in Table 1.

It can be seen in the table that the angle between the Burgers vector and the dislocation line is 90° in [001]-bending, indicating the pure edge character of the generated dislocation. For the loading direction of [112], the corresponding angles are determined to be 70.5° and 29.5°, corresponding to the edge-dominant and screw-dominant mixed dislocations, respectively. Likewise, this characteristic angle is determined as 35.3° and 19.5° for [111]-bending, corresponding to the screw-dominant mixed dislocations, while it equals 0° in [110]-bending, indicating the pure screw character. As can be seen, the dislocation types determined by the geometric method show good agreement with the DXA results. This geometric method effectively identifies the dislocation types produced by bending and reveals a prominent dependence on the loading direction. The loading direction determines the dislocation characteristics by governing the orientation of the nanowire surfaces subjected to bending stress; such surface further dictates geometrically the dislocation line direction. Moreover, it is also noted that these screw-predominant dislocations in [111]- and [110]-bending preferentially nucleate from the compressed lower surface rather than the tensile upper surface of the nanowire. A possible explanation for such asymmetry lies in the fact that the core of the ⟨111⟩ screw dislocation in the B2 intermetallic spreads into three non-parallel planes of the ⟨111⟩ zone, similar to that in BCC metals [40]. Compressive stress reduces atomic spacing and lowers the nucleation energy barrier of screw dislocations, thereby facilitating dislocation nucleation and propagation.

#### 3.3.3. Dislocation Density

**Figure 10:**
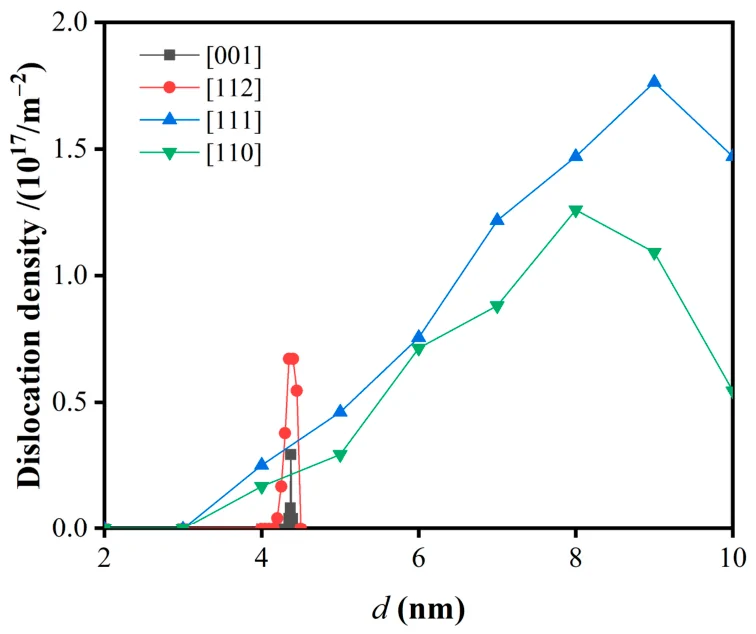
Evolutions of the dislocation density in the ⟨110⟩-oriented B2-FeAl nanowire upon bending along different directions.

During plastic deformation, dislocation multiplication leads to changes in their type, quantity and distribution, which in turn affect the mechanical behavior of the nanowire. To further investigate the influence of the loading direction on the deformation behavior of the nanowire, the dislocation density has also been quantified. Since the bending forces lie within the longitudinal symmetry plane of the nanowire, it gives rise to a symmetric in-plane bending deformation. Under such loading conditions, the dislocation density is defined as the number of dislocations intersecting the longitudinal symmetry plane per unit area. The number of dislocations *n* could be extracted directly by DXA analysis. The area *S* of the longitudinal symmetry plane is calculated as *S* = *Lh*, where *L* is the nanowire length, and *h* denotes its height. The dislocation density is thus determined as *ρ* = *n*/*S*. Figure 10 presents the evolution of dislocation density as a function of displacement for various loading directions. As can be seen, the overall trend of these curves is similar for all loading directions, which generally exhibits an initial increase followed by a drop. However, significant differences exist in the curve profiles related to different loading directions. For [001]- and [112]-bending, the dislocation density rises rapidly to a low peak within a small displacement range and subsequently drops sharply to zero; this evolution of dislocation density results from the brittle fracture of the nanowire. In contrast, the dislocation density increases steadily with deformation up to a significantly higher peak under the [111]- and [110]-bending, which is associated with the sustained plastic deformation in the nanowire.

Under bending, a certain density of geometrically necessary dislocations (GNDs) is required to accommodate the strain gradient due to the condition of geometric compatibility. According to Nye [49] and Ashby [50], the GND density is proportional to the bending curvature of the nanowire, which is given as

(1) $$\rho_{\mathrm{GND}}=\frac{\kappa_{p}}{b}$$

where *κ*_p_ denotes the plastic curvature and b is the component of the Burgers vector parallel to the nanowire axis. The plastic curvature is further determined as *κ*_p_ = 2*θ*_P_*/L*, in which *θ*_P_ is the plastic bending angle and *L* is the nanowire length. The plastic bending angle can be estimated by subtracting the elastic bending angle from the total bending angle, i.e., *θ*_P_ = *θ* − *θ*_e_, where *θ* denotes the total bending angle and *θ*_e_ is the elastic bending angle. The instantaneous total bending angle *θ* can be evaluated directly at each selected timestep; particularly, it equals to *θ*_e_ during the elastic deformation stage. In the present study, we have $b=\frac{\sqrt{3}}{2}a\cos\varphi$ for the 1/2<111> dislocations, where *a* = 0.2990 nm is the lattice constant of B2-FeAl alloy and *φ* represents the angle between the Burgers vector and the nanowire axis. The different bending behavior with respect to the loading directions can be further understood from the perspective of GND. According to Equation (1), Table 2 presents the comparison of the actual dislocation density and the GND density related to different loading directions at some representative displacements. Under [001]- and [112]-bending, the dislocation density is significantly lower than the required GND density for accommodating the lattice curvature, failing to meet the compatibility condition associated with bending. As a result, plastic deformation becomes highly localized, which induces stress concentration and ultimate brittle fracture. Contrary to the [001]- and [112]-bending, the dislocation density under bending along [111] and [110] directions is comparable to the GND density. This allows for the effective accommodation of the geometric mismatch caused by bending through dislocation activity, thereby retarding strain localization and enabling sustained bending plasticity. Notably, the dislocations produced by [111]- and [110]-bending stop within the nanowire rather than escape to the surfaces, finally serving as “geometrically necessary” dislocations.

## 4. Discussion

The above results indicate that the yielding mechanism in the ⟨110⟩-oriented B2-FeAl nanowire upon bending is governed by dislocation nucleation from the surface, which appears to be independent of the loading direction. However, the deformation behavior and failure modes of the nanowire show a strong dependence on the loading direction. Specifically, the FeAl nanowire fails in a brittle manner when bent along [001] and [112] directions, as manifested by negligible plastic deformation and the flat fracture surface. In contrast, [111]- and [110]-bending endow the nanowire with excellent ductility, as evidenced by the large bending curvature. The related deformation behavior is also reflected by the evolution of the dislocation density during bending, where minimal dislocation density is produced by [001]- and [112]-bending, where as it increases steadily to a high peak value when subjected to [111]- and [110]-bending. It appears that loading along [001] favors brittle fracture, whereas improved ductility is achieved as the loading direction approaches [110]. This contrasting behavior demonstrates a brittle-to-ductile transition in the B2-FeAl nanowire when the loading direction changes from [001] to [112], then to [111], and finally to [110].

The brittle-to-ductile transition with respect to the loading direction can be attributed to its influence on the dislocation characteristics. In the present study, it has been shown that near-[001] bending was governed by the slip of dislocations with predominant edge components, while screw-dominant dislocations gradually prevail as the loading direction trends towards [110]. Such distinction in dislocation types determines the cross-slip propensity, thereby controlling the tendency for brittle fracture versus plastic flow in the nanowire. For the edge-dominant dislocations generated in near-[001]bending, the glide motions are restricted to a unique slip plane, and cross-slip is virtually impossible. The motions of these edge-dominant dislocations would be hindered by the extensive stress gradient as they move towards the nanowire interior. As a consequence, stress concentrates severely within the nanowire and rapidly approaches the fracture strength of the nanowire, eventually triggering brittle fracture with little plastic deformation. On the other hand, the dislocations produced by near-[110] bending can switch their glide planes via cross-slip due to their screw characteristics. The cross-slip processes of these screw-like dislocations allow the nanowire to relax stress concentrations, enabling sustained plastic deformation. Consequently, the nanowire can undergo uniform plastic deformation with a large bending angle, accompanied by an increase in dislocation density up to a high level. As can be seen, the loading direction dictates the dislocation characteristics and, in turn, their cross-slip propensity, which fundamentally determines the bending behavior and failure modes, thereby leading to the brittle-to-ductile transition in the B2-FeAl nanowire.

Notably, the cross-slip propensity of dislocations emerges as the critical factor that governs the ductility of the B2-FeAl nanowire. This governing role of cross-slip in the brittle-to-ductile transition is also recognized in bulk BCC metals, which have recently been shown to be governed by the relative mobility ratio of screw-to-edge dislocations [51,52]. This mobility ratio between screw and edge dislocations is correlated with the efficiency of the Frank–Read dislocation source. A higher glide speed of screw dislocations enhances the efficiency of Frank–Read sources, thereby improving the capacity to carry plastic deformation. On the other hand, such Frank–Read dislocation sources become inactive as the sample dimensions decrease to the nanoscale. The absence of dislocation sources shifts the plastic deformation mechanism from dislocation multiplication in bulk metals to surface-nucleated dislocation motions in the nanowire. As revealed in the above results, the screw-dominant dislocations govern the ductility of nanowires by cross-slip-mediated strain delocalization, which differs substantially from the mobility ratio-dependent mechanism observed in the bulk counterparts. Accordingly, this work extends the traditional understanding of the brittle-to-ductile transition from a purely temperature-governed phenomenon to a behavior controlled by loading direction. In turn, the loading conditions may serve as a key parameter to achieve tunable mechanical properties, enabling either on-demand brittleness via edge-dominant dislocation pile-ups or exceptional bending plasticity via screw-dominant dislocation cross-slip.

## 5. Conclusions

In this work, we have investigated the bending behavior of a ⟨110⟩-oriented B2-FeAl nanowire with different loading directions using MD simulations. The main results are summarized as follows:

(1) The ⟨110⟩-oriented B2-FeAl nanowire yields under bending by nucleation of dislocations, which is independent of the loading direction. However, changes in loading direction result in a brittle-to-ductile transition in the nanowire. Specifically, bending along directions of [001] and [112] leads to brittle failure of the nanowire, while a shift of the loading direction toward [111] and [110] produces prominent plastic deformation in the nanowire with large bending curvature.

(2) The brittle-to-ductile transition, with respect to the loading direction, may be understood through the analysis of its influence on the dislocation characteristics. Whereas [001]- and [112]-bending produce edge-dominant dislocations with limited capability for cross-slip, resulting in stress concentration and subsequent brittle fracture, plastic deformation in [111]- and [110]-bending is mediated by the slip of dislocations with predominant screw characteristics, whose cross-slip process effectively relaxes stress localizations and allows sustained plastic flow within the nanowire.

(3) A physical model based on the geometric constraints of bending deformation has been constructed and shown to reasonably characterize the variation in dislocation types with respect to different loading directions. This model provides a concise and effective theoretical method for interpreting the anisotropy of bending plasticity in nanowires under bending.

## Figures and Tables

**Table 1:** Predictions of dislocation types in the ⟨110⟩-oriented FeAl nanowire under bending along different directions using the geometric model.

Loading Direction	Slip Plane	Line Direction	Angle Between *b* and *l* (°)	Dislocation Type
[001]	$\left(\bar{1}12\right)$	[110]	90	Pure edge
[112]	(011)	$\left[11\bar{1}\right]$	70.5	Edge-dominant
	$\left(10\bar{1}\right)$	$\left[1\bar{3}1\right]$	29.5	Screw-dominant
[111]	(011)	$\left[01\bar{1}\right]$	35.3	Screw-dominant
	$\left(10\bar{1}\right)$	$\left[1\bar{2}1\right]$	19.5	Screw-dominant
[110]	(011)	$\left[1\bar{1}1\right]$	0	Pure screw
	$\left(10\bar{1}\right)$	$\left[1\bar{1}1\right]$	0	Pure screw

**Table 2:** Comparisons of actual dislocation density and GND density in the ⟨110⟩-oriented FeAl nanowire under bending along different loading directions.

Loading Direction	Displacement	Dislocation Density (10^17^·m^−2^)	GND Density (10^17^·m^−2^)
[001]	*d* = 4.4 nm	0.013	0.042
[112]	*d* = 4.3 nm	0.15	0.23
[111]	*d* = 6.0 nm	0.76	0.75
	*d* = 7.0 nm	1.25	1.20
[110]	*d* = 6.0 nm	0.71	0.68
	*d* = 7.0 nm	0.88	0.87

## Data Availability

Data can be made available upon reasonable request.
